# Quantitative SPECT imaging of ^155^Tb and ^161^Tb for preclinical theranostic radiopharmaceutical development

**DOI:** 10.1186/s40658-024-00682-8

**Published:** 2024-09-14

**Authors:** Helena Koniar, Scott McNeil, Luke Wharton, Aidan Ingham, Michiel Van de Voorde, Maarten Ooms, Sathiya Sekar, Cristina Rodríguez-Rodríguez, Peter Kunz, Valery Radchenko, Arman Rahmim, Carlos Uribe, Hua Yang, Paul Schaffer

**Affiliations:** 1 TRIUMF, Life Sciences Division, 4004 Wesbrook Mall, Vancouver, BC V6T 2A3 Canada; 2 Department of Physics and Astronomy, University of British Columbia, 6224 Agricultural Road, Vancouver, BC CAN V6T Canada; 3 Institute for Nuclear Medical Applications Science, SCK CEN, Boeretang 200, Mol, BE 2400 Canada; 4 Faculty of Pharmaceutical Sciences, University of British Columbia, 2405 Wesbrook Mall, Vancouver, BC V6T 1Z3 Canada; 5 TRIUMF, Accelerator Division, 4004 Wesbrook Mall, Vancouver, BC V6T 2A3 Canada; 6 Department of Chemistry, Simon Fraser University, 8888 University Drive, Burnaby, BC V5A 1S6 Canada; 7 Department of Chemistry, University of British Columbia, 2036 Main Mall, Vancouver, BC V6T 1Z1 Canada; 8 Department of Radiology, University of British Columbia, 2775 Laurel Street, Vancouver, BC V5Z 1M9 Canada; 9 BC Cancer Research Centre, Department of Integrative Oncology, 675 W 10th Ave, Vancouver, BC V5Z 1L3 Canada; 10 Functional Imaging, BC Cancer Agency, 600 West 10th Avenue, Vancouver, BC V5Z 4E6 Canada

**Keywords:** Terbium-155, Terbium-161, Radiopharmaceutical therapy, Theranostic pair, Preclinical imaging, Quantitative SPECT

## Abstract

**Background:**

Element-equivalent matched theranostic pairs facilitate quantitative in vivo imaging to establish pharmacokinetics and dosimetry estimates in the development of preclinical radiopharmaceuticals. Terbium radionuclides have significant potential as matched theranostic pairs for multipurpose applications in nuclear medicine. In particular, ^155^Tb (t_1/2_ = 5.32 d) and ^161^Tb (t_1/2_ = 6.89 d) have been proposed as a theranostic pair for their respective applications in single photon emission computed tomography (SPECT) imaging and targeted beta therapy. Our study assessed the performance of preclinical quantitative SPECT imaging with ^155^Tb and ^161^Tb. A hot rod resolution phantom with rod diameters ranging between 0.85 and 1.70 mm was filled with either ^155^Tb (21.8 ± 1.7 MBq/mL) or ^161^Tb (23.6 ± 1.9 MBq/mL) and scanned with the VECTor preclinical SPECT/CT scanner. Image performance was evaluated with two collimators: a high energy ultra high resolution (HEUHR) collimator and an extra ultra high sensitivity (UHS) collimator. SPECT images were reconstructed from photopeaks at 43.0 keV, 86.6 keV, and 105.3 keV for ^155^Tb and 48.9 keV and 74.6 keV for ^161^Tb. Quantitative SPECT images of the resolution phantoms were analyzed to report inter-rod contrast, recovery coefficients, and contrast-to-noise metrics.

**Results:**

Quantitative SPECT images of the resolution phantom established that the HEUHR collimator resolved all rods for ^155^Tb and ^161^Tb, and the UHS collimator resolved rods ≥ 1.10 mm for ^161^Tb and ≥ 1.30 mm for ^155^Tb. The HEUHR collimator maintained better quantitative accuracy than the UHS collimator with recovery coefficients up to 92%. Contrast-to-noise metrics were also superior with the HEUHR collimator.

**Conclusions:**

Both ^155^Tb and ^161^Tb demonstrated potential for applications in preclinical quantitative SPECT imaging. The high-resolution collimator achieves < 0.85 mm resolution and maintains quantitative accuracy in small volumes which is advantageous for assessing sub organ activity distributions in small animals. This imaging method can provide critical quantitative information for assessing and optimizing preclinical Tb-radiopharmaceuticals.

## Background

There are four terbium isotopes of clinical interest due to their versatile applications in nuclear medicine with suitable half-lives, diverse decay modes, and emission energies [1, 2]. ^149^Tb (t_1/2_ = 4.12 h), ^152^Tb (t_1/2_ = 17.5 h), ^155^Tb (t_1/2_ = 5.32 d), and ^161^Tb (t_1/2_ = 6.89 d) have applications in alpha therapy, PET, SPECT, and β^-^/Auger therapy, respectively [3–5]. Matched element-equivalent theranostic pairs have been investigated for many isotopes including ^43^Sc/^44^Sc/^47^Sc [6, 7], ^64^Cu/^67^Cu [8], ^86^Y/^90^Y [9], ^123^I/^124^I/^131^I [10], ^132^La/^135^La [11], ^209^At/^211^At [12], ^203^Pb/^212^Pb [13], and ^225^Ac/^226^Ac [14, 15]. Element-equivalent pairs benefit from identical radiolabelling conditions, pharmacokinetics, and biodistribution, facilitating quantitative assessment and optimization in radiopharmaceutical development and enabling accurate dosimetry estimates for clinical applications [16, 17].

**Fig. 1:**
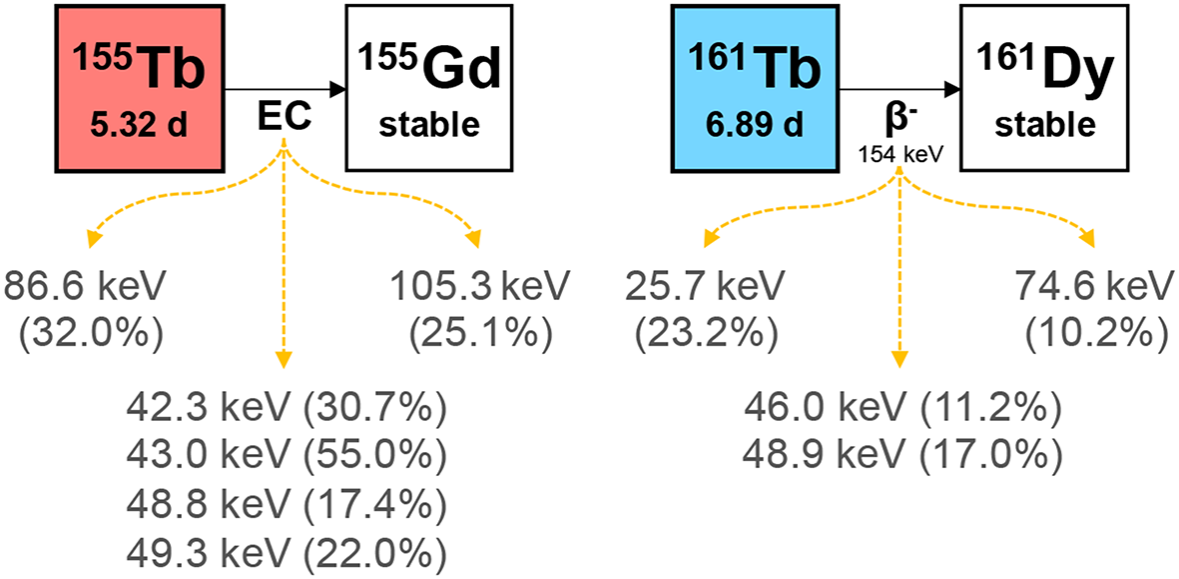
Decay characteristics of ^155^Tb (*left*) and ^161^Tb (*right*). X-ray and gamma photon emissionswith absolute intensity > 10% are shown. Half-lives and decay radiation properties were extracted from Livechart (IAEA Nuclear Data Section) based on the Nov 2023 ENSDF snapshot

Of the four terbium isotopes described above, ^155^Tb and ^161^Tb have been identified as a promising theranostic pair with ^155^Tb serving as a diagnostic isotope and ^161^Tb as a therapeutic isotope (Fig. 1). ^155^Tb decays purely via electron capture to stable ^155^Gd emitting photons with energies of 42.3 keV (30.7%), 43.0 keV (55.0%), 48.7 keV (17.4%), 49.3 keV (22.0%), 86.6 keV (32.0%), and 105.3 keV (25.1%) [18], making it a promising candidate for single photon emission computed tomography (SPECT) imaging. Quantitative SPECT imaging enables diagnostic information on disease spread and assists in pre-therapy dosimetry calculations [17, 19]. Furthermore, ^155^Tb emits an average of 13.9 Auger electrons and 0.77 conversion electrons per decay which could be leveraged for Auger electron therapy [20, 21]. ^161^Tb decays via β^-^-decay to stable ^161^Dy with decay characteristics suitable for treating metastatic disease, emitting 154 keV mean energy β^-^ particles and an average of 10.9 Auger electrons and 1.4 conversion electrons per decay [21, 22]. In addition to its therapeutic emissions, ^161^Tb also has photon emissions that enable SPECT imaging with energies at 25.7 keV (23.2%), 46.0 keV (11.2%), 48.9 keV (17.0%), and 74.6 keV (10.2%) [23]. The higher energy 48.9 keV and 74.6 keV gamma emissions are particularly useful for small animal preclinical SPECT imaging.

While ^155^Tb and ^161^Tb have been proposed as matched theranostic pair, given their individual decay characteristics they could also act as standalone theranostic agents with ^155^Tb for SPECT imaging and Auger electron therapy and ^161^Tb for SPECT imaging and β^-^-therapy. Several routes for ^155^Tb and ^161^Tb production are currently under investigation for their feasibility in larger scale clinical quantities while maintaining high yield and radionuclidic purity [24, 25]. Although there are standing challenges in optimizing large scale routine production, this pair of Tb isotopes remain very promising for clinical applications in nuclear medicine [3].

Previous studies have demonstrated the in vivo potential and capabilities of Tb isotopes [1]. SPECT imaging of Derenzo hot rod resolution phantoms with both ^155^Tb and ^161^Tb has been previously conducted and revealed excellent spatial resolution [1, 22, 26, 27]. However, metrics on the limits of resolution, quantitative accuracy, and optimal image reconstruction methods for these Tb-based SPECT imaging systems have not yet been well characterized and require further investigation. Quantitative imaging at both the preclinical and clinical stages can assist in estimating dosimetry and optimizing therapy outcomes.

Preclinical studies have explored in vivo imaging with several promising radiopharmaceuticals including [^155/161^Tb]Tb-cm09 [1], [^161^Tb]Tb-PSMA-617 [28], [^161^Tb]Tb-DOTA-TOC [29], [^155^Tb]Tb-DOTA-TOC [27], [^155/161^Tb]Tb-crown-TATE [20], and [^155/161^Tb]Tb-crown-αMSH [30]. Further preclinical work involving ^161^Tb-radiopharmaceuticals has assessed the in vivo stability [31] and biodistribution profiles [32] but often lacks corresponding quantitative SPECT imaging data.

In-human clinical invesitagtions with ^161^Tb have remained limited relative to the β^-^ emitter ^177^Lu, in part, due to production challenges however, there have been promising initial outcomes. Clinical protocols for ^161^Tb SPECT/CT imaging have been established with high resolution capabilities [33]. In two patients with metastatic neuroendocrine neoplasms, [^161^Tb]Tb-DOTA-TOC SPECT/CT imaging demonstrated the feasibility of visualizing even small metastases with relatively low injected activities [34]. Following a single treatment with [^161^Tb]Tb-PSMA-617, one patient with advanced metastatic castration-resistant prostate cancer exhibited impressive partial remission, even with disease progression following extensive [^177^Lu]Lu-PSMA-617 therapy [35]. Additionally, post-therapy SPECT/CT imaging of [^161^Tb]Tb-PSMA-617 is feasible for visualizing its distribution in the targeted lesions and non-target organs [36]. The ongoing VIOLET Phase I/II clinical trial aims to evaluate the therapeutic efficacy and safety of [^161^Tb]Tb-PSMA-I&T for metastatic prostate cancer [37]. Post-therapy quantitative SPECT/CT imaging was also evaluated over a range of activities and imaging times to produce suitable imaging for clinical review [38]. Further, in silico dosimetry studies have indicated that ^161^Tb may be a better candidate than ^177^Lu for irradiating single cancer cells and micrometastasis [39].

Evidently there has been tremendous interest in the development of theranostic pairs with ^155^Tb and ^161^Tb. As production and purification methods improve Tb availability for preclinical and clinical studies, quantitative SPECT imaging protocols are necessary to further optimize the development pipeline of novel Tb-radiopharmaceuticals. In this work, we assess and evaluate the imaging performance of ^155^Tb and ^161^Tb with a preclinical SPECT/CT scanner and establish a SPECT/CT protocol for quantitative imaging. Element-equivalent matched theranostic pairs provide accurate biodistribution and dosimetry estimates, and this quantitative pharmacokinetic information is essential in optimizing the efficacy of radiopharmaceuticals in preclinical settings.

## Methods

### ^155^Tb and ^161^Tb production and activity quantification

^155^Tb was produced at TRIUMF (Vancouver, Canada) with its Isotope Separation and Acceleration (ISAC) facility. 480 MeV protons were used to irradiate a tantalum target, resulting in a heterogeneous ion beam of spallation products that are mass separated by the principles of isotope separation on-line (ISOL) [40, 41]. The A/q = 155 beam was implanted onto an aluminum implantation target with a thin ammonium chloride salt layer. ^155^Er (t_1/2_ = 5.3 m), ^155^Ho (t_1/2_ = 48 m), and ^155^Dy (t_1/2_ = 9.9 h) were co-implanted with the ^155^Tb activity, however, all these isotopes decay into ^155^Tb via β^+^/EC-decay during a 5-day cool-down period after end of beam delivery. The salt layer on the implantation target was dissolved with small volumes of water (< 500 µL) prior to transfer to a sample vial for further dilution into imaging phantoms. Production methods are described only briefly here and are fully described in Fiaccabrino et al. (2021) [41]. Yield measurements from all production runs are documented in the ISAC Yield Database [42].

^161^Tb was produced at SCK CEN (Mol, Belgium) with its BR2 reactor [22, 43]. A highly enriched ^160^Gd target (97.5% enrichment, as ^160^Gd_2_O_3_) was irradiated for 7 days using a high thermal neutron flux of 3 × 10^14^ neutrons/cm^2^/s. Neutron activation of the enriched gadolinium (^160^Gd) target yields the short-lived ^161^Gd (t_1/2_ = 3.66 m) which decays into ^161^Tb via β^-^-decay. The target was dissolved with high purity 1 M HNO_3_. ^161^Tb was purified from the ^160^Gd target material and ^161^Dy decay product with an automated system of solid-phase extraction columns. Purification methods are described briefly here and are fully described in McNeil et al. (2022) [32].

Small samples of the ^155^Tb and ^161^Tb solutions were collected for activity quantifications. The activity was measured by gamma spectroscopy with a high-purity geranium (HPGe) detector (Canberra Industries, Meriden, CT) and analyzed with the *Genie* 2000 software package (Canberra Industries, Meriden, CT). Activity measurements have 8% estimated uncertainties, compounded from volume measurement and calibration source uncertainties [44]. For this study, activity concentrations of 21.8 ± 1.7 MBq/mL of ^155^Tb and 23.6 ± 1.9 MBq/mL of ^161^Tb were used for point source calibration and resolution phantom imaging.

### SPECT image acquisition and reconstruction

The performance of ^155/161^Tb-based imaging was assessed with the VECTor (Versatile Emission Computed Tomography) microSPECT/CT (MILabs, Utretch, Netherlands). Its imaging capacities have been well described by Goorden et al. (2013) [45]. Briefly, the VECTor scanner consist of three stationary NaI gamma camera detectors placed in a triangle with pinhole collimators designed to collect data in a central field of view (CFOV) [46]. With a translation stage bed, phantoms and animals can be moved throughout imaging acquisition to collect scintillation events in the pinholes from different regions in the scanned subject [47].

While MILabs produces many collimators compatible with the VECTor scanner, two collimators were evaluated for their scanning performance: a high energy ultra high resolution (HEUHR) collimator and an extra ultra high sensitivity (UHS) collimator. The HEUHR collimator is ideal for high energy photons (> 350 keV) and high spatial resolution [48] while the UHS collimator is optimized for low energy photons (< 350 keV) and high sensitivity [49]. The HEUHR collimator is made of tungsten with an inner diameter of 44 mm and consists of 48 four-pinhole clusters with 0.35 mm diameter pinholes. When assessed with ^99m^Tc and ^18^F, its peak sensitivity was 2,800 cps/MBq and 2,899 cps/MBq and the reconstructed spatial resolution was 0.5 mm and 0.8 mm, respectively. The UHS collimator is made of lead with an inner diameter of 46 mm and consists of 54 conical 2.0 mm diameter pinholes. When assessed with ^99m^Tc, the peak sensitivity was 13,080 cps/MBq and reconstructed spatial resolution was 0.85 mm. Both collimators were evaluated to characterize ^155^Tb and ^161^Tb image quality and its applications for in vivo quantitative SPECT imaging. Robertson et al. [50] and Koniar et al. [14] serve as a basis for this work in our assessment of Tb-based imaging [14, 50].

**Fig. 2:**
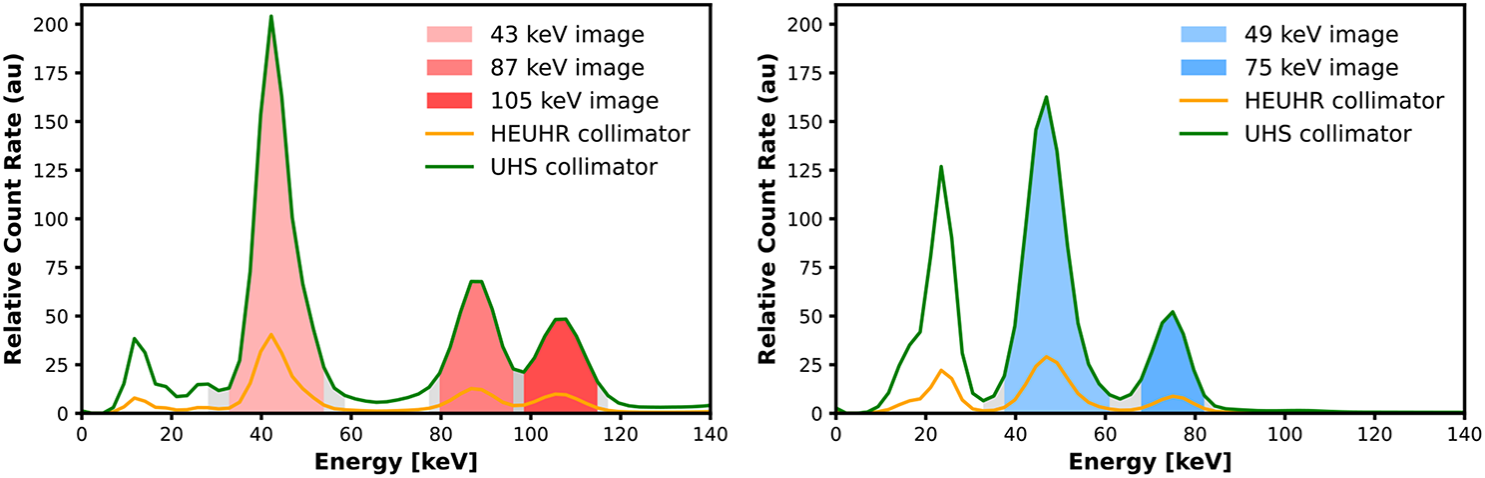
Photon energy spectrum of ^155^Tb (*left*) and ^161^Tb (*right*)

Data was acquired in list mode and sorted into 512 energy bins with 2.34 keV width. The FOV was set to 54 mm × 54 mm × 140 mm for both the UHS and HEUHR collimator for all image acquisitions. The images were reconstructed from photopeaks at 43.0 keV, 86.6 keV, and 105.3 keV for ^155^Tb and at 48.9 keV and 74.6 keV for ^161^Tb (see Fig. 2). The 43.0 keV photopeak for ^155^Tb and the 48.9 keV photopeak for ^161^Tb are the result of multiple photon emissions within a close energy range. More precisely, the 43.0 keV photopeak is a combination of 42.3 keV (30.7%), 43.0 keV (55.0%), 48.7 keV (17.4%), 49.3 keV (22.0%) emissions and the 48.9 keV photopeak is a combination of 46.0 keV (11.2%) and 48.9 keV (17.0%) emissions. For simplicity and clarity, reconstructions from these photopeak are referred to by their most probable photon emission, namely the 43.0 keV and 48.9 keV emissions. Figure 2. demonstrates the relative count rate, or sensitivity normalized to the activity concentration and scan time, for both the HEUHR and UHS collimators as a function of photon energy and corresponds well with published photon intensities seen in Fig. 1.

A pixel-based ordered subset expectation maximization (OSEM) iterative reconstruction algorithm (16 subsets, 6 iterations) was used to reconstruct the images with 0.4 mm^3^ voxel size. This number of subsets and iterations (96 MLEM-equivalent iterations) was determined to optimize the contrast-to-noise ratio for most resolution phantom images when considering different combinations of isotopes (^155^Tb and ^161^Tb) and collimators (UHS and HEUHR). The contrast-to-noise ratio calculation is presented in further detail below. The reconstruction algorithm was maintained for all images to investigate the effect of photopeak and collimator choice on resulting SPECT image quality. SPECT images were corrected for background and scatter by the triple energy window method and for attenuation by CT image co-registration (Table 1). The width of energy windows was selected to include mostly primary photons counted by the detectors. The width of the upper and lower energy background windows was set with 2.5 background weight as specified by the manufacturer. SPECT images were re-sampled to CT images with 0.169 mm^3^ voxels. Non-uniform attenuation correction was employed post-reconstruction with a CT-based attenuation map [51].

**Table 1:** Energy window parameters for SPECT image reconstruction

Isotope	Photopeak energy	Photopeak window	Upper/Lower window
^155^Tb	43.0 keV	50%	4.3%
	86.6 keV	18%	3.1%
	105.3 keV	15%	3.2%
^161^Tb	48.9 keV	50%	4.9%
	74.6 keV	20%	3.6%

### Point source calibration

Quantitative SPECT images were obtained via calibration factors determined through SPECT scans of activity point source phantoms for each collimator and isotope combination as described by the manufacturer. Activity concentration was also determined independently via gamma spectroscopy. Point source phantoms consisted of Eppendorf tubes filled with decay corrected ^155^Tb or ^161^Tb activity as listed in Table 2. Scan time, and total counts in the photopeak and background windows for all combinations of reconstruction photopeak and collimator are presented in Table 2. Calibration factors were determined with volumes of interest centered inside the point source phantom SPECT reconstructions for all collimator and photopeak reconstruction combinations. All reconstructed SPECT/CT images were analyzed with Python (v3.8.8) scripts.

**Table 2:** Point source phantom scan time, activity decay-corrected to time of imaging, and counts in each reconstruction window for each isotope and collimator combination

		UHS				HEUHR			
Isotope	Photopeak energy	Scan time	Activity concentration (MBq/mL)	Photopeak counts (millions)	Background counts (millions)	Scan time	Activity concentration (MBq/mL)	Photopeak counts (millions)	Background counts (millions)
^155^Tb	43.0 keV	15 min	47.1 ± 3.8	35.83	2.61	30 min	46.6 ± 3.7	14.00	0.96
	86.6 keV			13.80	2.06			5.00	0.75
	105.3 keV			10.61	2.15			4.17	0.74
^161^Tb	48.9 keV	30 min	23.2 ± 1.9	32.56	1.35	30 min	23.5 ± 1.9	6.01	0.29
	74.6 keV			8.99	0.82			1.57	0.19

### Resolution phantom

**Fig. 3:**
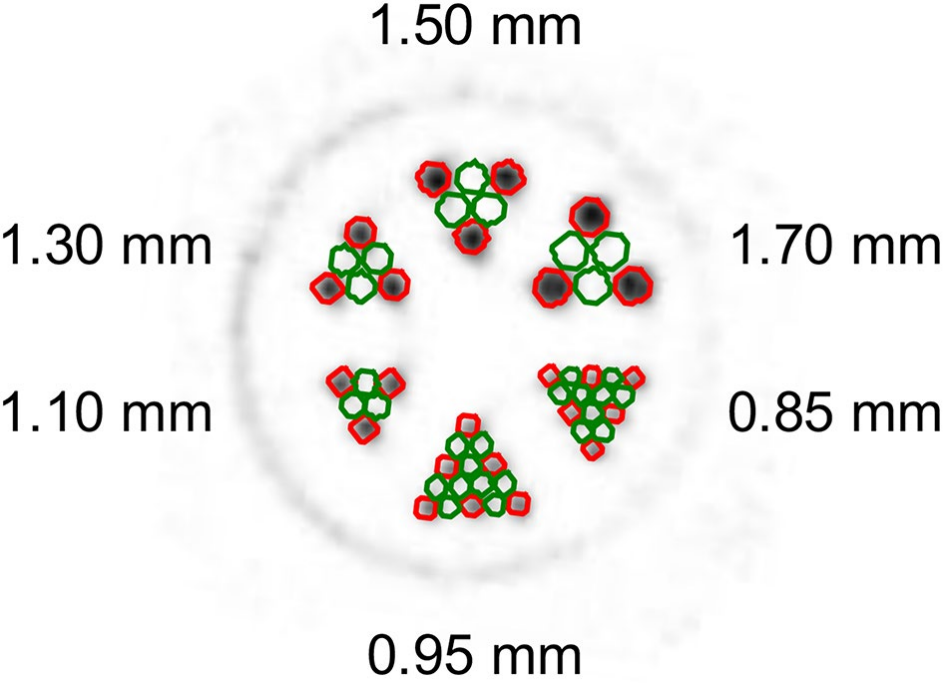
Sample of a cross section of the resolution phantom with 0.85–1.70 mm diameter rods. Red and green circles represent the hot ($\:{h}_{d}$) and background ($\:{b}_{d}$) ROIs, resepectively, for each rod diameter $\:d$

A hot rod resolution phantom with thin rod clusters of diameters 0.85 mm, 0.95 mm, 1.10 mm, 1.30 mm, 1.50 mm, and 1.70 mm was used to assess the resolution of the VECTor imaging system (see Fig. 3). The resolution phantom was filled with decay corrected activities of ^155^Tb (21.4 ± 1.7 MBq/mL for the UHS scan; 20.9 ± 1.7 MBq/mL for the HEUHR scan) or with ^161^Tb (23.4 ± 1.9 MBq/mL for the UHS scan; 23.5 ± 1.9 MBq/mL for the HEUHR scan). Due to the high activity concentrations of ^155^Tb and ^161^Tb, images were acquired rapidly, with 60 min total acquisition time for all isotope and collimator combinations. Quantitative SPECT images of the resolution phantoms were analyzed to report spatial resolution, quantitative accuracy, and noise metrics as defined by Walker et al. (2014) [52]. Briefly here, circular ROIs with 0.8 times the physical diameter were placed inside the rods and in between the rods to measure the mean value and repeated across 30 planes for an axial thickness of 5.1 mm.

Inter-rod contrast ($\:{C}_{d}$) quantifies resolvability in small hot regions. Inter-rod contrast for rods with diameter $\:d$ was defined as:

$$\:{C}_{d}=\frac{\stackrel{-}{{h}_{d}}-\stackrel{-}{{b}_{d}}}{\stackrel{-}{{h}_{d}}}$$

where $\:\stackrel{-}{{h}_{d}}$ is the mean value inside the rods and $\:\stackrel{-}{{b}_{d}}$ is the mean value in between the rods. The ideal inter-rod contrast is 1, and rods with inter-rod contrast greater than 0.2 were visually resolvable.

The recovery coefficient (RC) quantifies the accuracy of the apparent activity concentrations. The RC was defined as:

$$\:RC=\frac{\stackrel{-}{{h}_{d}}}{{h}_{0}}$$

where $\:{h}_{0}$ is the activity concentration determined independently via gamma spectroscopy. The ideal RC is also 1.

The measure for quantifying the variability between ROI mean values for rods with diameter $\:d$ was defined as:

$$\:{N}_{d}=\frac{\sqrt{{\sigma\:}_{{h}_{d}}^{2}+{\sigma\:}_{{b}_{d}}^{2}}}{\overline{RO{I}_{d}}}$$

where $\:{\sigma\:}_{{h}_{d}}^{2}$ and $\:{\sigma\:}_{{h}_{d}}^{2}$is the variance in $\:{h}_{d}$ and $\:{b}_{d}$, calculated over 6 of the 30 image slices to reduce covariance between adjacent planes, and $\:\stackrel{-}{RO{I}_{d}}$ is the mean value from all ROIs ($\:{h}_{d}$ and $\:{b}_{d}$) with rod diameter $\:d$. The contrast-to-noise ratio (CNR) was defined as $\:{C}_{d}/{N}_{d}$ as another measure for image quality.

### Impact of activity concentration on image quality metrics

Spatial resolution and noise metrics were also assessed in SPECT images reconstructed with fewer counts, to simulate lower activity concentrations or shorter scan times more commonly seen in preclinical in vivo imaging. Count reductions randomly select a portion of the list mode acquired data to be ignored during image reconstruction and emulate images acquired with lower activity concentrations. Images were reconstructed with 20%, 5%, 1%, and 0.5% of the total counts to investigate the effects on image quality.

## Results

### Resolution phantom

**Fig. 4:**
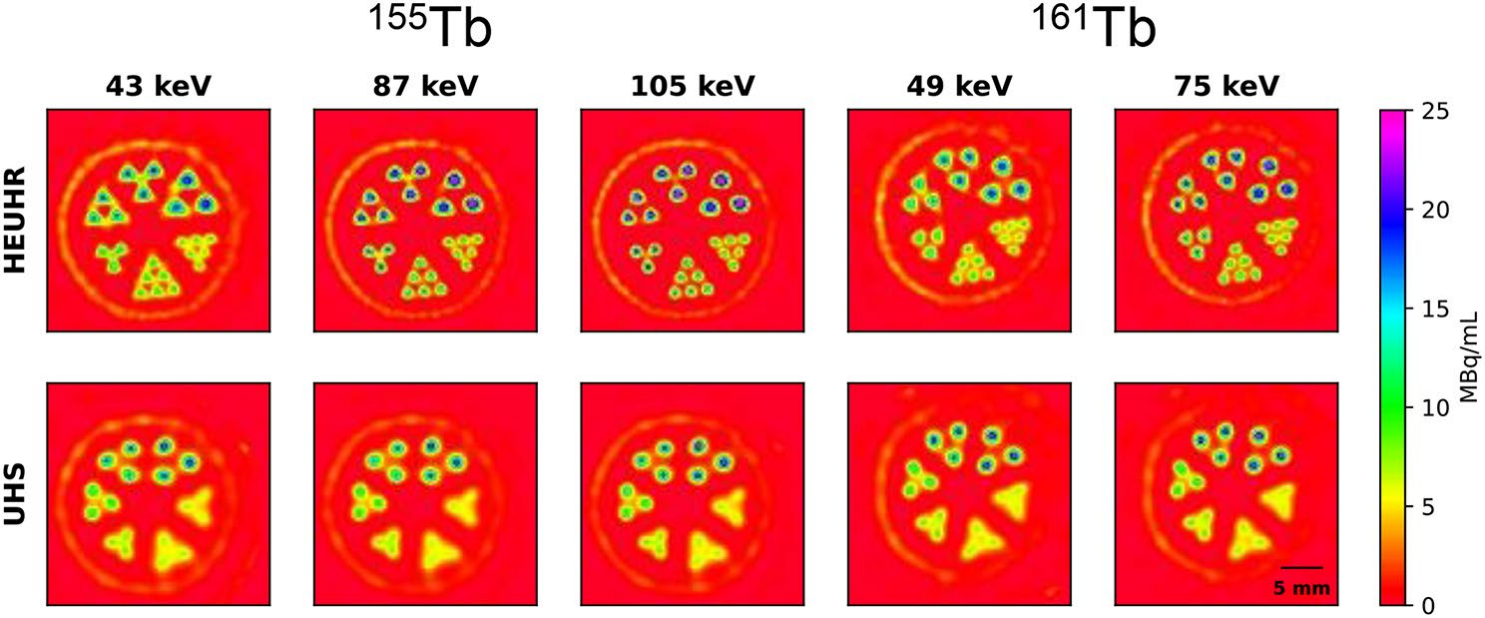
Quantitative SPECT images of the resolution phantom reconstructed with various photopeak windows after acquisition with the HEUHR and UHS collimators. No post-reconstruction smoothing was applied

Figure 4 shows quantitative SPECT images of the resolution phantom acquired with the HEUHR and UHS collimators with various photon emission photopeak reconstructions. Qualitatively, it is apparent that the HEUHR collimator resolves rods better than the UHS collimator for all ^155^Tb and ^161^Tb emission photopeak reconstructions. Further, it is visually evident that the HEUHR collimator resolves all rods, indicating a resolution of < 0.85 mm for all reconstructions.

### Inter-rod contrast and resolution limit

**Fig. 5:**
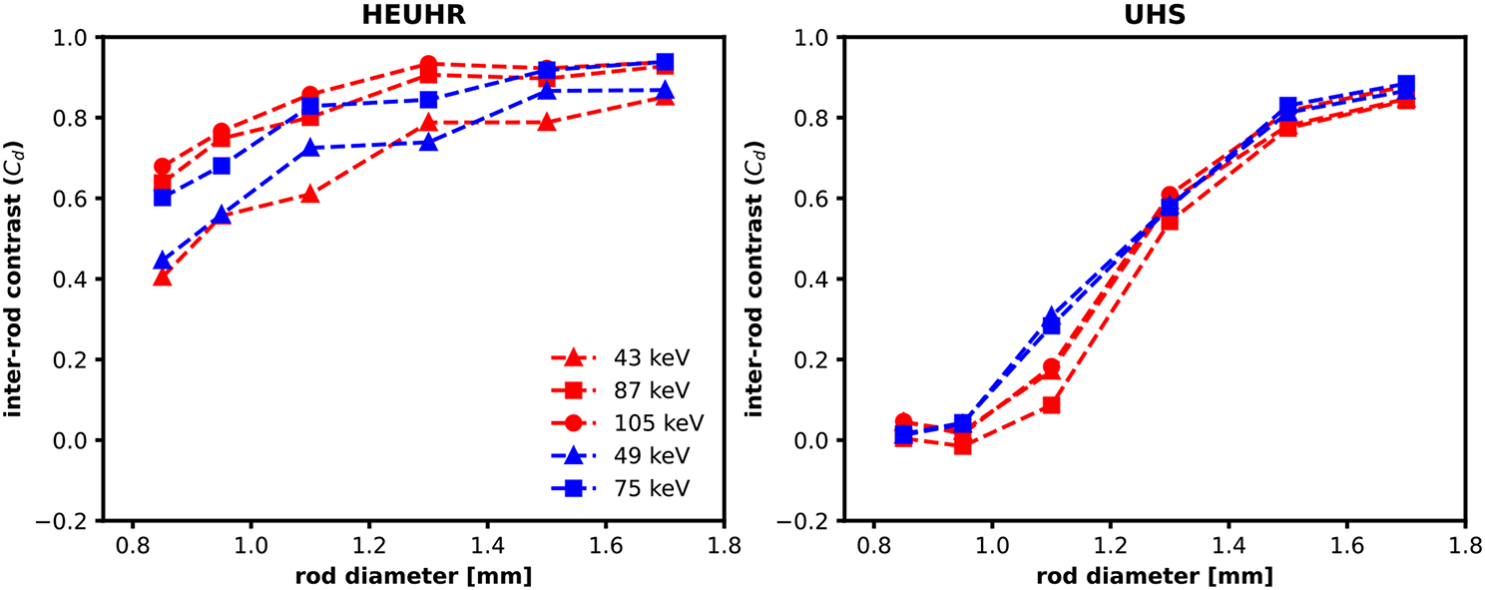
Inter-rod contrast (C_d_) measurements from SPECT images of the resolution phantom acquired with the HEUHR (*left*) and UHS (*right*) collimators with ^155^Tb in red and ^161^Tb in blue

Inter-rod contrast measurements (Fig. 5) determine the minimum resolvability of SPECT images of the resolution phantom. Images acquired with the HEUHR collimator had inter-rod contrast values > 40% for all rods and photopeak reconstructions, indicating that the imaging system’s minimum resolvability is < 0.85 mm. Images reconstructed from higher energy photopeaks (105 keV for ^155^Tb and 75 keV for ^161^Tb) had higher inter-rod contrast values than those reconstructed from lower energy photopeaks (45 keV and 87 keV for ^155^Tb and 49 keV for ^161^Tb). This is seen qualitatively in Fig. 4 by less blurring, or lower apparent activity, in between the rods for SPECT images reconstructed from higher energy photopeaks. However, in images acquired with the UHS collimator, only rods with diameters ≥ 1.10 mm for ^161^Tb and ≥ 1.30 mm for ^155^Tb were resolvable. Unlike with the HEUHR collimator, the inter-rod contrast metrics from images acquired with the UHS collimator did not vary significantly with different photopeak energy reconstructions.

**Fig. 6:**
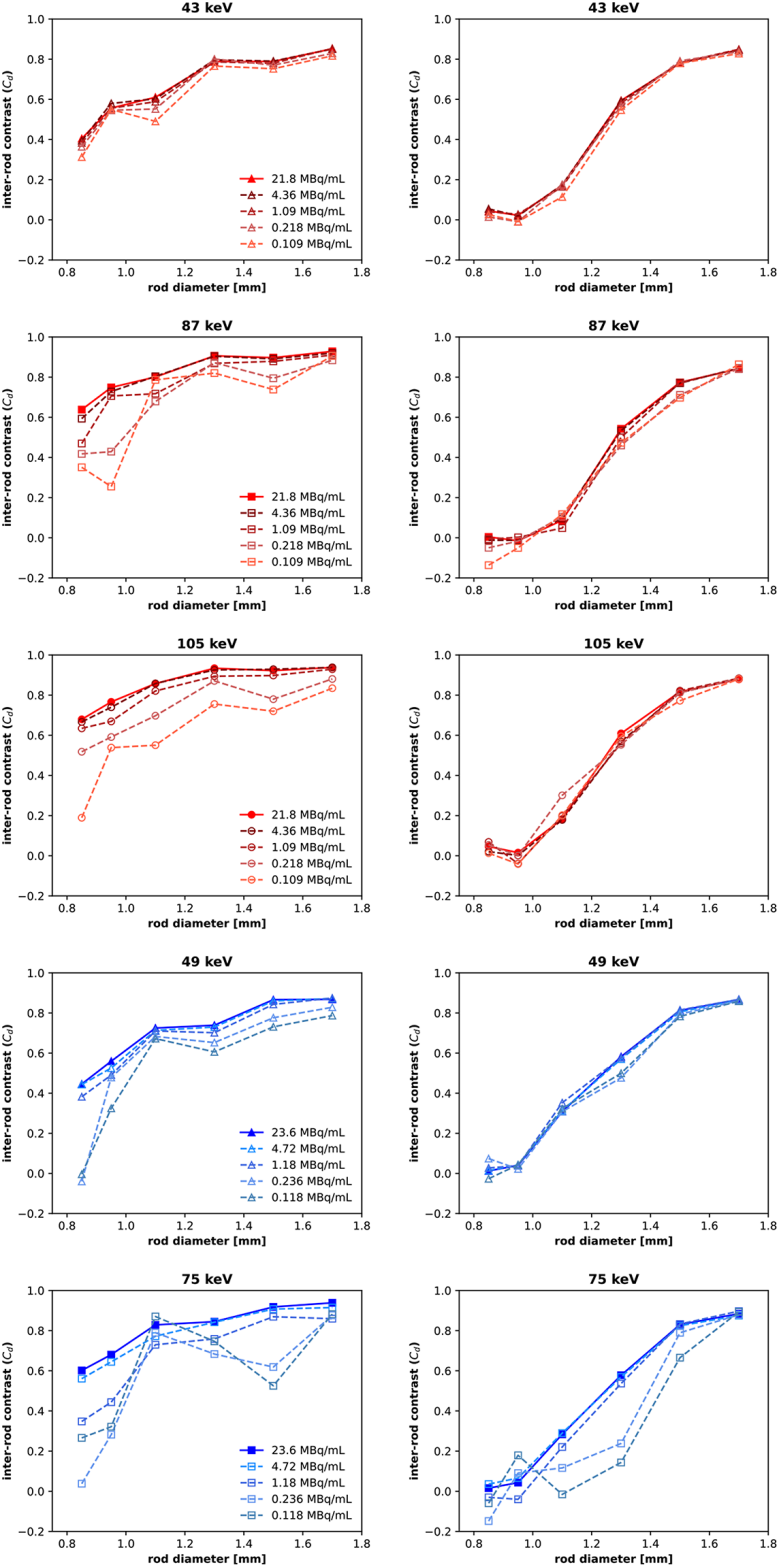
Inter-rod contrast measurements from SPECT images of the resolution phantom with reduced activity concentrations, simulated by reconstructing with 20%, 5%, 1%, and 0.5% of the total acquired counts. SPECT images were acquired with the HEUHR (*left*) and UHS (*right*) collimators with ^155^Tb in red and ^161^Tb in blue

At lower activity concentrations, the inter-rod contrast with the HEUHR collimator remained consistent down to 1 MBq/mL (5% total counts) maintaining the same overall resolvability seen in the full count SPECT images (see Fig. 6). Inter-rod contrast with the UHS collimator was less affected by lower total counts than the HEUHR collimator as system resolution remained consistent for all activity concentrations ≥ 0.1 MBq/mL (0.5% total counts).

### Recover coefficient and quantitative accuracy

**Fig. 7:**
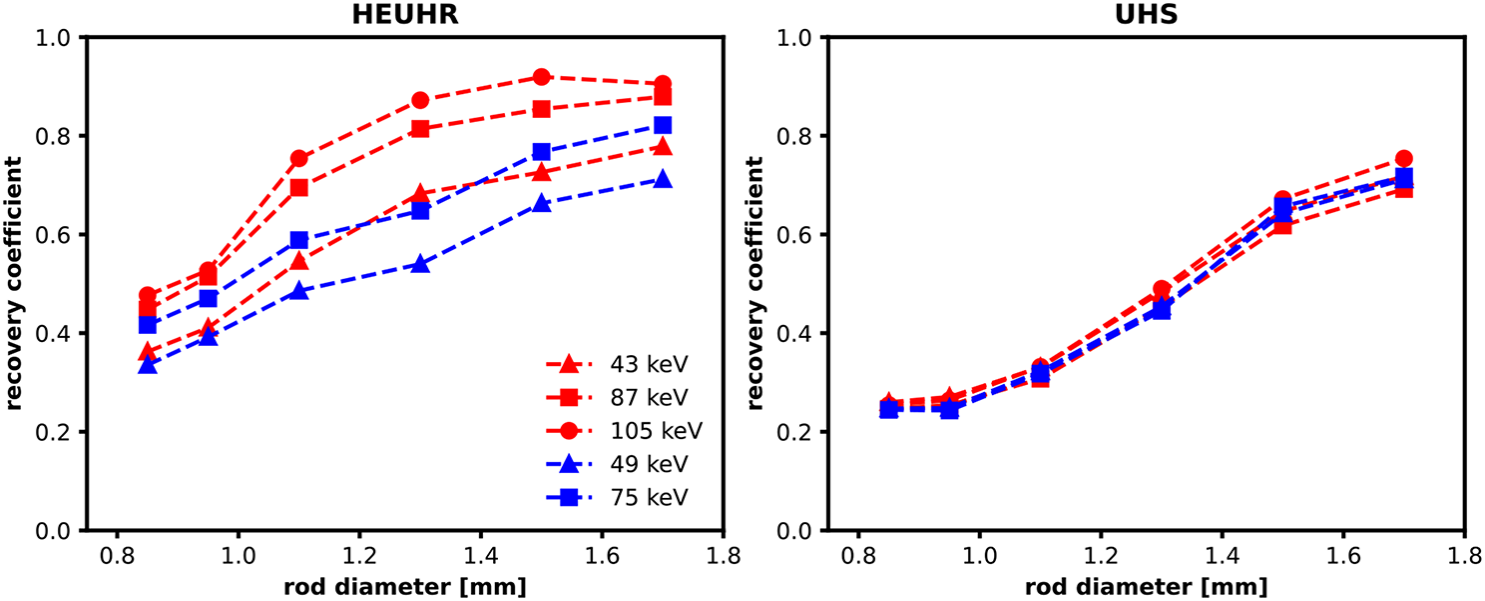
Recovery coefficients from SPECT images of the resolution phantom acquired with the HEUHR (*left*) and UHS (*right*) collimators with ^155^Tb in red and ^161^Tb in blue

Figure 7 shows the RC metrics for evaluating the quantitative accuracy of SPECT images of the resolution phantom. For all photopeak reconstructions of ^155^Tb and ^161^Tb, the HEUHR collimator outperformed the UHS collimator with higher RC values indicative of better quantitative accuracy. For the 1.70 mm diameter rods, RC values ranged between 71 and 91% with the HEUHR collimator and between 69 and 75% with the UHS collimator. Like the inter-rod contrast value results presented above, SPECT images acquired with the HEUHR collimator and reconstructed from higher energy photopeaks had better quantitative accuracy. The best overall quantitative accuracy was seen in the SPECT images of the ^155^Tb-filled resolution phantom reconstructed from the high energy 105 keV photopeak; with RC values > 75% for rods with diameters ≥ 1.1 mm and maximum value of 92% quantitative accuracy. Similarily, SPECT images of the ^161^Tb-filled resolution phantom showed better quantitative accuracy with its 75 keV reconstruction than the lower energy 49 keV reconstruction, with higher RC values demonstrated for all rod diameters. The RC values for images acquired with the UHS collimator were relatively independent from the photopeak energy.

**Fig. 8:**
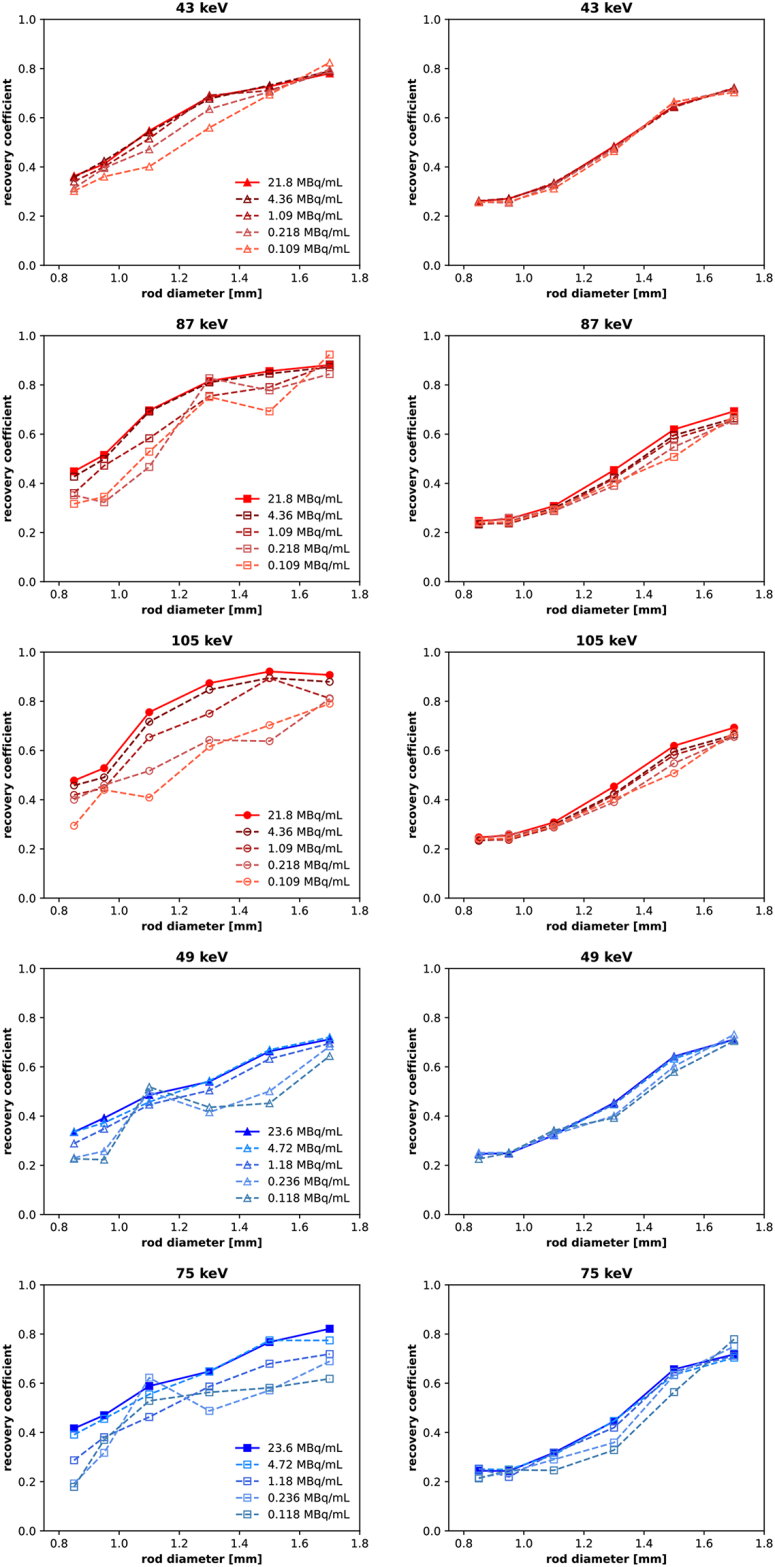
Recovery coefficients from SPECT images of the resolution phantom with reduced activity concentrations, simulated by reconstructing with 20%, 5%, 1%, and 0.5% of the total acquired counts. SPECT images were acquired with the HEUHR (*left*) and UHS (*right*) collimators with ^155^Tb in red and ^161^Tb in blue

At lower activity concentrations, RC values become more variable and are generally lower than those seen in the full count SPECT images (see Fig. 8). The HEUHR collimator maintains consistent quantitative accuracy for activity concentrations ≥ 1MBq/mL while the UHS collimator maintains performance with activity concentrations ≥ 0.1 MBq/mL.

### Contrast-to-noise metrics

**Fig. 9:**
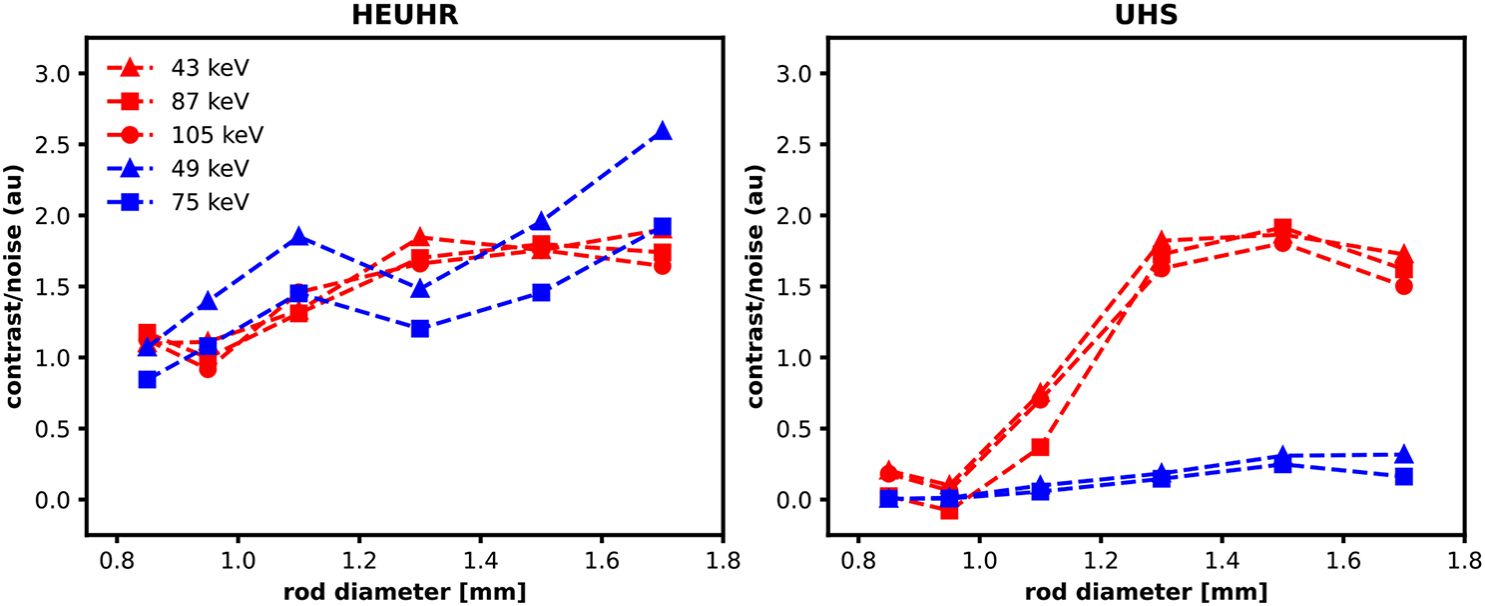
Contrast-to-noise ratios from SPECT images of the resolution phantom acquired with the HEUHR (*left*) and UHS (*right*) collimators with ^155^Tb in red and ^161^Tb in blue

**Fig. 10:**
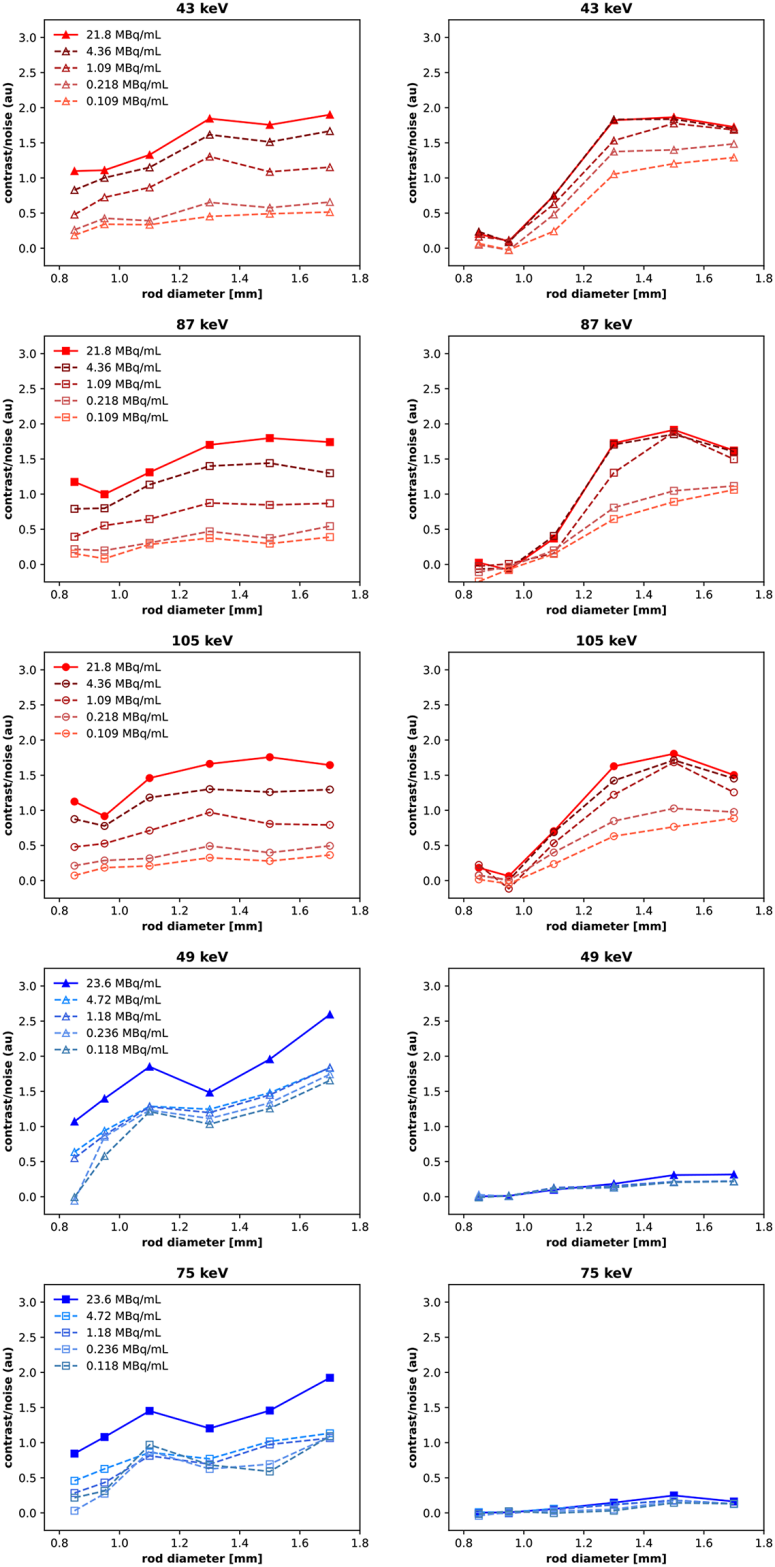
Contrast-to-noise ratios from SPECT images of the resolution phantom with reduced activity concentrations, simulated by reconstructing with 20%, 5%, 1%, and 0.5% of the total acquired counts. SPECT images were acquired with the HEUHR (*left*) and UHS (*right*) collimators with ^155^Tb in red and ^161^Tb in blue

CNR values are presented in Fig. 9. Images acquired with the HEUHR collimator generally had a higher CNR with increasing rod diameter, although the CNR plateaued for ^155^Tb images. The lower energy photopeak reconstructions (43 keV for ^155^Tb and 49 keV for ^161^Tb) slightly outperformed the higher energy photopeak reconstructions. Images acquired with the UHS collimator demonstrated notably better CNRs with the ^155^Tb-filled phantom than the ^161^Tb-filled phantom. The reconstructed photopeak energy did not have a meaningful effect on CNR values for images acquired with the UHS collimator. For both collimators, CNRs are positively correlated to the activity concentration as seen by decreasing CNRs with lower total counts (see Fig. 10).

## Discussion

In this work, we have evaluated the performance of two different collimators for preclinical SPECT imaging of ^155^Tb and ^161^Tb by quantifying their image characteristics and limitations with a hot rod resolution phantom.

The HEUHR collimator universally achieves a resolution of < 0.85 mm, making it suitable for in vivo imaging in mice. At this scale, sub-organ localization of Tb-labelled radiopharmaceuticals can be discerned. This level of resolution is consistent with findings from previous studies that evaluated this collimator [48]. With the HEUHR collimator, SPECT imaging with of ^225^Ac’s progeny isotopes ^221^Fr (218 keV) and ^213^Bi (440 keV) achieved comparable resolution limits below 0.85 mm [50]. Similarly, SPECT imaging of ^226^Ac with its 158 keV and 230 keV gamma emissions also had resolvability < 0.85 mm with the same collimator [14].

Images acquired with the HEUHR collimator exhibited better resolved rods and noticeably higher inter-rod contrast when reconstructed from higher energy photopeaks. This is likely due to lower energy photons are having a higher probability of scattering and thus requiring wider photopeak energy windows (as shown in Table 1). The addition of scattered photons in the primary photopeak window results in blurring and degraded rod profiles leading to less contrast between rods [53]. Considering the low energy (< 140 keV) of photons emitted from ^155^Tb and ^161^Tb, this result is consistent with the findings of previous work with this imaging system. The improved inter-rod contrast could be attributed to the image reconstruction system matrix provided by the manufacturer which is optimized for 140 keV energy photons. We have observed that as the photon energy approaches 140 keV, the inter-rod contrast improves. The effect of photon energy on inter-rod contrast has also been seen with higher energy (> 140 keV) photons from the ^225^Ac decay chain, where the 218 keV (^221^Fr) image was better than the 440 keV (^213^Bi) image [50] and for ^226^Ac where the 158 keV image was better than the 230 keV image [14]. Despite being designed for collimating high energy photons, this work indicates that the HEUHR collimator is well suited to maintain its resolution for photon emissions with energies ≥ 40 keV with sufficient activity concentrations (see Fig. 6). The resolution and quantitative accuracy demonstrated in this preclinical imaging system benefits from the relatively small proportion of attenuated photons in small animals. These imaging cabapilities might not translate to clinical scanners since there will be significantly more scatter and attenuation of low energy photon emissions in humans. However, the ability to quantify preclinical images should not be understated in the optimization and development of preclinical Tb-radiopharmaceuticals.

The UHS collimator yielded resolvability of rods ≥ 1.10 mm for ^161^Tb and ≥ 1.30 mm for ^155^Tb, consistent with previous work on this imaging system. With the UHS collimator, SPECT images of ^225^Ac’s progeny ^221^Fr (218 keV) resolved rods ≥ 1.30 mm [50] and images of ^226^Ac resolved rods ≥ 1.30 mm and ≥ 1.50 mm for the 158 keV and 230 keV images, respectively [14]. Inter-rod contrast with the UHS collimator was less affected by photopeak energy than the HEUHR collimator which could be attributed to the higher sensitivity of the UHS collimator which permits a higher relative count rate than the HEUHR collimator (Fig. 2) and lessens the effect of including scattered photons in the primary photopeak window. While the UHS collimator does not have as powerful resolution capabilities as the HEUHR collimator, it is less affected by lower activity concentrations. This is especially important in considerations for in vivo imaging applications with generally lower activity concentrations and shorter scan times than those seen in a phantom imaging study.

Quantitative accuracy in small volumes is important to assess in a resolution phantom to understand the impact of partial volume effects on in vivo activity measurements. The RC values from images acquired with the HEUHR collimator showed the best quantitative accuracy with the high energy 105 keV gamma from ^155^Tb demonstrating the highest RC values overall for all rod diameters. The UHS collimator does not perform as well in terms of maintaining quantitative accuracy in small volumes. This is expected given the results of the inter-rod contrast measurements which limit the inherent system resolution to ≥ 1.10 mm for ^161^Tb and ≥ 1.30 mm for ^155^Tb. In future in vivo applications, it is important to account for these RC values to estimate true activity concentrations more accurately from the apparent activity measured in small volumes from quantitative SPECT images.

In high-count SPECT images, the HEUHR collimator provides high CNR between small hot volumes with both ^155^Tb and ^161^Tb. The UHS collimator provides notably higher CNRs for ^155^Tb than ^161^Tb. As such, ^155^Tb is the stronger candidate for imaging in the development of preclinical Tb-radiopharmaceuticals. When considering in vivo imaging scenarios, it is important to note that low-count SPECT images will exhibit lower CNRs in small hot volumes such as the adrenal glands or gallbladder in mice.

Overall, the HEUHR collimator provides better spatial resolution and quantitative accuracy in SPECT images of the resolution phantom than the UHS collimator. However, the UHS collimator maintains better image quality in images reconstructed from simulated low activity concentrations ≥ 0.1 MBq/mL. For preclinical in vivo imaging applications, a collimator should be selected based on anticipated activity concentrations from injected activity levels and anticipated uptake in tumours or organs of interest. In cases where high-count SPECT images can be acquired the HEUHR collimator is superior and in cases where only low-count SPECT images can be obtained the UHS collimator is better suited. Images reconstructed from higher energy photopeaks demonstrated better overall image quality than those reconstructed from their counterpart lower energy photopeaks for some activity concentration and collimator combinations. The flexibility of image reconstruction from multiple different gamma emissions photopeaks is a strong advantage of SPECT imaging with ^155^Tb and ^161^Tb. It is quite feasible to reconstruct several images from different photon energies after an in vivo scan acquisition to optimize both quantitative and qualitative image quality metrics. This ability to adapt image reconstruction parameters is a strength to this set of isotopes, in addition to its element-equivalent in vivo behaviour as theranostic isotopes. These advantages can facilitate the preclinical development of radiopharmaceuticals in a more efficient manner.

## Conclusion

Quantitative SPECT imaging of ^155^Tb and ^161^Tb can be performed with high spatial resolution using the preclinical small-animal SPECT/CT VECTor scanner. A high-resolution collimator is superior for quantitative accuracy in high count SPECT images; however, a high sensitivity collimator provides better qualitative images in low count SPECT images. Both ^155^Tb and ^161^Tb have multiple photon emissions that provide adaptability in image reconstruction protocols to optimize image quality for the development of novel preclinical Tb-based radiopharmaceuticals.

## Data Availability

The datasets generated and analysed in the current study are available from the corresponding author on reasonable request.

## Abbreviations

CNR: Contrast to noise ratio
CT: Computed tomography
EC: Electron capture
ENSDF: Evaluated nuclear structure data file
HEUHR: High energy ultra high resolution
HPGe: High-purity geranium
ISAC: Isotope separation and acceleration
ISOL: Isotope separation on-line
MLEM: Maximum likelihood expectation maximization
OSEM: Ordered subset expectation maximization
RC: Recovery coefficient
ROI: Region of interest
SPECT: Single photon emission computed tomography
UHS: Extra ultra high sensitivity
VECTor: Versatile emission computed tomography
